# More airway smooth muscle in males versus females in a mouse model of asthma: A blessing in disguise?

**DOI:** 10.1113/EP091236

**Published:** 2023-06-21

**Authors:** Rebecka Gill, Andrés Rojas‐Ruiz, Magali Boucher, Cyndi Henry, Ynuk Bossé

**Affiliations:** ^1^ Institut Universitaire de Cardiologie et de Pneumologie de Québec (IUCPQ), Université Laval Département de médecine Québec Canada

**Keywords:** airway hyperresponsiveness, airway remodelling, allergic inflammation

## Abstract

Mouse models are helpful in unveiling the mechanisms underlying sex disparities in asthma. In comparison to their female counterparts, male mice are hyperresponsive to inhaled methacholine, a cardinal feature of asthma that contributes to its symptoms. The physiological details and the structural underpinnings of this hyperresponsiveness in males are currently unknown. Herein, BALB/c mice were exposed intranasally to either saline or house dust mite once daily for 10 consecutive days to induce experimental asthma. Twenty‐four hours after the last exposure, respiratory mechanics were measured at baseline and after a single dose of inhaled methacholine that was adjusted to trigger the same degree of bronchoconstriction in both sexes (it was twice as high in females). Bronchoalveolar lavages were then collected, and the lungs were processed for histology. House dust mite increased the number of inflammatory cells in bronchoalveolar lavages to the same extent in both sexes (asthma, *P* = 0.0005; sex, *P* = 0.96). The methacholine response was also markedly increased by asthma in both sexes (e.g., *P* = 0.0002 for asthma on the methacholine‐induced bronchoconstriction). However, for a well‐matched bronchoconstriction between sexes, the increase in hysteresivity, an indicator of airway narrowing heterogeneity, was attenuated in males for both control and asthmatic mice (sex, *P* = 0.002). The content of airway smooth muscle was not affected by asthma but was greater in males (asthma, *P* = 0.31; sex, *P* < 0.0001). These results provide further insights regarding an important sex disparity in mouse models of asthma. The increased amount of airway smooth muscle in males might contribute functionally to their greater methacholine response and, possibly, to their decreased propensity for airway narrowing heterogeneity.

## Abstract (highlights)

- **What is the central question of this study?**
  The lung response to inhaled methacholine is reputed to be greater in male than in female mice. The underpinnings of this sex disparity are ill defined.
- **What is the main finding and its importance?**
  We demonstrated that male airways exhibit a greater content of airway smooth muscle than female airways. We also found that, although a more muscular airway tree in males might contribute to their greater responsiveness to inhaled methacholine than females, it might also curb the heterogeneity in small airway narrowing.

## 1 INTRODUCTION

Sex influences the prevalence and severity of asthma (Chowdhury et al., 2021; Fuseini & Newcomb, 2017; Melgert et al., 2007; Zein & Erzurum, 2015). The mechanisms underlying sex disparities in asthma are difficult to study in humans. Firstly, asthma inception, development and severity are influenced by an interacting host of genetic factors (Berube & Bosse, 2014) and inhaled offending triggers that vary in concentration and constitutional composition over time (Sheehan et al., 2017; Szram & Cullinan, 2012). The impact of these interactions also depends on the timing (e.g., age, menstrual cycle, pregnancy) and sequence of exposure throughout life (Tang et al., 2021), in addition to the diet (Williams et al., 2022), the lung and gut microbiota (Barcik et al., 2020; Tang et al., 2021), the presence of obesity (Tooba & Wu, 2022) and other co‐morbidities (Cazzola et al., 2022; Teodorescu et al., 2015), the current and historical intake of medications (Tan et al., 2021), the air temperature (Zhou et al., 2022), and levels of anxiety (Ye et al., 2021), physical activity (Kuder et al., 2021), sunshine (Alfredsson et al., 2020) and ambient oxygen (altitude) (de Nijs et al., 2020; Vinnikov et al., 2016). Secondly, investigations of humans in vivo rely on non‐invasive methods that lack precision (Bates & Irvin, 2003). Finally, detailed investigations at the cell or tissue scales in humans are limited by the small selection and restricted amount of excised cells and tissues (Looi et al., 2011), usually derived from a small number of individuals and often studied *ex vivo* after being transformed by culturing conditions (Baldassi et al., 2021). Thus, isolating the contribution of sex in asthma might seem like a simple task, but it is not.

Animal models allow us to measure with more invasive and substantially more precise tools (Bates & Irvin, 2003) the effect of a single intervention, while controlling all other confounders. Animal models are also useful to fill gaps regarding tissue accessibility. Although murine models can always be criticized in terms of their relevance to human asthma pathogenesis, they recapitulate some features that are reminiscent of human asthma, including, inter alia, a T‐helper 2 type of inflammation and hyperresponsiveness to methacholine.

As a result, murine models have been used for decades to study sex disparities in asthma (Blacquiere et al., 2010; Corteling & Trifilieff, 2004; E‐Lacerda et al., 2020; Hayashi et al., 2003; Lauzon‐Joset et al., 2020; Melgert et al., 2010, 2005; Schaefer et al., 2020; Seymour et al., 2002; Takeda et al., 2013; Weiss et al., 2021; Zhao et al., 2019). It is now established that although female mice are more susceptible to the development of pulmonary inflammation upon exposure to allergens (E‐Lacerda et al., 2020; Hayashi et al., 2003; Laffont et al., 2017; Lauzon‐Joset et al., 2020; Melgert et al., 2010, 2005; Schaefer et al., 2020; Seymour et al., 2002; Takeda et al., 2013; Weiss et al., 2021; Zhao et al., 2019), male mice are more responsive to methacholine (Boucher, Dufour‐Mailhot et al., 2022; Card et al., 2006, 2007). The latter finding is not universal (Berndt et al., 2011), but is consistently reported in many mouse strains (Berndt et al., 2011; Card et al., 2006). Notably, it is marked in BALB/c mice (Boucher, Dufour‐Mailhot et al., 2022; Card et al., 2006, 2007), the mouse strain most widely used to study experimental asthma (Carroll et al., 2023).

Many mechanisms were put forward to explain the propensity of females for pulmonary allergic inflammation, including decreases in: regulatory T cells (Melgert et al., 2005); group 2 innate lymphoid cells (Laffont et al., 2017); oestrogen (Lauzon‐Joset et al., 2020); alternatively activated macrophages (Melgert et al., 2010); and an interleukin (IL)‐13 response to IL‐33 (Zhao et al., 2019). Another very recent study characterized further the obvious sex disparities in the lung inflammatory response to allergens using two different strains of mice (Mostafa et al., 2022). Therefore, the differing inflammatory responses to allergens between sexes have been well studied and will not be discussed in detail herein. In contrast, the mechanisms contributing to the greater methacholine response in males than in females have been less studied (Boucher, Dufour‐Mailhot et al., 2022; Card et al., 2006, 2007).

In the present study, experimental asthma was induced in female and male BALB/c mice to characterize further the sex difference in the methacholine response. A particular feature of the study was the methacholine challenge, during which the dose was adjusted according to the degree of responsiveness in each sex. The goal was to determine whether the many contributors to the methacholine response (e.g., lung stiffening, bronchoconstriction and airway narrowing heterogeneity) differ between sexes for a matched degree of bronchoconstriction. Structural components of the airway wall relevant to the methacholine response were also compared between sexes. The results provided insights regarding the mechanisms underlying the enhanced methacholine response in male mice, but also highlighted a role of the airway smooth muscle (ASM) in counteracting an important contributor to hyperresponsiveness.

## 2 MATERIALS AND METHODS

### 2.1 Ethical approval

All procedures were approved by the Committee of Animal Care of *Université Laval* in accordance with guidelines of the Canadian Council on Animal Care (protocols 2018‐005‐4 and 2022‐977‐1).

### 2.2 Mice

Forty female and 40 male BALB/c mice (Charles River, Saint‐Constant, Canada) were tested at 8 weeks of age. They were provided with food and water ad libitum at all times.

### 2.3 Protocol to induce experimental asthma

**FIGURE 1:**
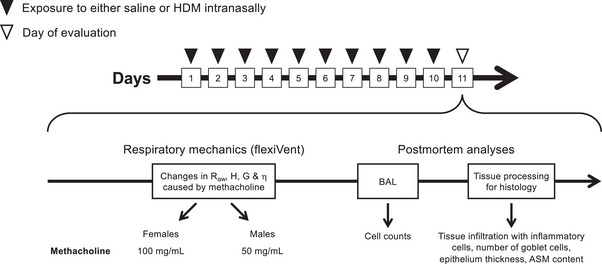
Schematic diagram depicting the sequence of interventions in the mouse study. See Materials and Methods for further details. Abbreviations: ASM, airway smooth muscle; BAL, bronchoalveolar lavages; *G*, tissue resistance; *H*, lung elastance; HDM, house dust mite; *R*_aw_, airway resistance; η, hysteresivity.

Mice of each sex were divided into two groups of 20 (Figure 1): one group exposed to 25 μL of saline (control mice) and one group exposed to 25 μL of 2 mg of house dust mite (HDM) extract (*Dermatophagoides pteronyssinus*; Greer, Lenoir, NC, USA) per millilitre of saline to induce pulmonary allergic inflammation (asthmatic mice) (Boucher, Henry, Dufour‐Mailhot et al., 2021, 2021; Sahu et al., 2010). The endotoxin concentration was 47.3 EU per milligram of HDM extract. They were exposed via intranasal instillation once daily under isoflurane anaesthesia for 10 consecutive days. All outcomes were measured 24 h after the last exposure.

### 2.4 Respiratory mechanics

Mice were weighed, and their respiratory mechanics were tested with the flexiVent (FX Module 2, SCIREQ, Montreal, QC, Canada) as described in our recent studies (Boucher, Dufour‐Mailhot et al., 2022; Boucher, Henry, Khadangi et al., 2021; Khadangi et al., 2021, 2022). Briefly, they were anaesthetized and put under systemic analgesia using ketamine (100 mg/kg) and xylazine (10 mg/kg). They were then tracheotomized and connected to the flexiVent through an 18‐gauge cannula, in a supine position. To avoid leakage, a surgical thread was passed around the trachea and tightened to seal the tracheal wall securely against the cannula. They were ventilated mechanically with room air at a tidal volume of 10 mL/kg with an inspiratory‐to‐expiratory time ratio of 2:3 at a breathing frequency of 150 breaths/min and with a positive end expiratory pressure of 3 cmH_2_O. Once the ventilation was started, muscle relaxation was induced by injecting 100 μL i.m. and 300 μL i.p. of pancuronium bromide (0.1 mg/kg) to avoid spontaneous breathing during the procedure. The heart rate was monitored throughout the experiment by ECG.

To assess respiratory mechanics, the lung was probed using an oscillometric perturbation called the Quick Prime‐3. More precisely, this perturbation was actuated twice at baseline and 15 times at 20 s intervals, starting 30 s after the end of the methacholine nebulization. Briefly, the Quick Prime‐3 is a composite input flow signal that enables the calculation of impedance of the respiratory system based on the resulting output pressure signal (Bates et al., 2011). Impedance was then analysed using a computational model called the constant phase model to deduce four metrics (Hantos et al., 1992): airway resistance (*R*_aw_), tissue resistance (*G*), tissue elastance (*H*) and hysteresivity (η); see Table 1 for further details.

**TABLE 1:** Physiological metrics used to measure the methacholine response in the present study.

Methods	Metrics	What it represents
The mouse lung is subjected to a multi‐frequency, small‐amplitude, composite sinusoidal flow perturbation, made of 13 sine waves of mutually prime frequencies with different amplitudes and phases. This is done at baseline and at regular intervals after the nebulized methacholine. After each Quick Prime‐3, the constant‐phase model (Hantos et al., 1992) is fitted to the calculated spectrum of impedance (i.e., the resistance and reactance measured at all tested frequencies). The constant‐phase model is: *Z*(ω) = *R*_aw_ + (*G* − i*H*)/ω^α^, where *Z* is impedance, ω is angular frequency, i is the imaginary unit, and α is 2/π arctangent of *H*/*G*. The changes in *R*_aw_, *G* and *H* are then used to monitor the response to methacholine.	*R*_aw_, airway resistance	*R*_aw_ is the resistance to airflow in conducting airways. Its increase in response to methacholine (Δ*R*_aw_) reflects bronchoconstriction
	*G*, tissue resistance	*G* is the resistance of the lung tissue. Its increase in response to methacholine (Δ*G*) reflects lung stiffening and airway narrowing heterogeneity (Lutchen et al., 1996)
	*H*, tissue elastance	*H* is the elastance of the whole lung. Its increase in response to methacholine (Δ*H*) reflects lung stiffening caused by tissue stiffening and/or small airway closure (Lundblad et al., 2007; Wagers et al., 2004)
	η, hysteresivity	η is the ratio of *G* over *H*. Its increase in response to methacholine (Δη) reflects a relatively greater gain in resistance than in elastance. It is a metric very sensitive to airway narrowing heterogeneity (Bates et al., 2009)

### 2.5 The methacholine challenge

For delivery of methacholine, the nebulizer for small particle size (Aeroneb Lab, Aerogen, Galway, Ireland) was operating for a duration of 10 s at a duty cycle of 50% under regular ventilation. As it is firmly established that males are substantially more responsive than females (Boucher, Dufour‐Mailhot et al., 2022; Card et al., 2006, 2007), the dose of methacholine was adjusted for sex. The goal was to give rise to a similar bronchoconstriction between sexes. Based on our previous study (Boucher, Dufour‐Mailhot et al., 2022), we predicted that a matched response should be obtained by doubling the methacholine concentration in females. The nebulized dose was thus set to a concentration of 50 mg/mL in males and 100 mg/mL in females. These doses also represent the maximal tolerable dose (i.e., the dose needed to generate a near maximal response without causing any death in asthmatic mice). The methacholine response was quantified by measuring the changes in oscillometry metrics (*H*, *G*, *R*_aw_ and η) from their baseline values to their peak values during the 5 min following the nebulization of the methacholine dose. The methacholine response was then compared between sexes and between control and asthmatic mice.

### 2.6 Euthanasia

Mice were killed by exsanguination immediately after the methacholine challenge.

### 2.7 Bronchoalveolar lavages

One millilitre of PBS was infiltrated into the lung through the trachea and aspirated to recover the bronchoalveolar lavages (BAL). This was repeated three times, and the recovered BAL were pooled. The total volume was recorded and centrifuged at 500*g* for 5 min. The supernatant was discarded and the pellet resuspended in 200 μL of PBS for control mice and 500 μL for asthmatic mice. Total cells in the BAL were stained with Crystal Violet and counted using a haemocytometer. Seventy‐five thousand cells were also cytospun and stained with modified May Grünwald–Giemsa to count the number of macrophages, lymphocytes, neutrophils and eosinophils.

### 2.8 Lung histology

Histology was performed as previously described (Khadangi et al., 2021; Mailhot‐Larouche et al., 2018) on the left lung. Briefly, the lung was excised and immersed in formalin for 24 h for fixation. The formalin was replaced by progressively increasing the ethanol concentration to dehydrate the tissue. The lung was then embedded in paraffin and cut transversely in 5‐μm‐thick sections. Sections were deposited on microscopic slides and stained with Haematoxylin and Eosin, Periodic acid–Schiff with Alcian Blue or Masson's trichrome. They were then scanned with a NanoZoomer Digital scanner (Hamamatsu photonics, Bridgewater, NJ, USA) at ×40 magnification.

Haematoxylin and Eosin staining was performed to evaluate the infiltration of inflammatory cells within the lung tissue. Fifteen non‐overlapping photomicrographs (1440 × 904 pixels) from three non‐contiguous lung sections were scored blindly from zero (no inflammation) to five (very severe inflammation) by one observer. The scores from each of the 15 photomicrographs were averaged to obtain one value per mouse, and values of all mice within one group were then compiled to obtain a mean per group.

Periodic acid–Schiff with Alcian Blue was used to count the number of goblet cells. All airways cut transversely in three non‐contiguous lung sections were analysed, representing 5–16 airways per mouse (mean and SD of 10.6 ± 2.5). The number of goblet cells within each airway was divided by the length of the basement membrane. The same staining was used to measure the thickness of the epithelium. All airways cut transversely and displaying a full circumference in three non‐contiguous lung sections were analysed, representing 3–16 airways per mouse (mean and SD of 9.9 ± 2.6). The epithelial thickness was analysed by measuring the area occupied by the epithelium divided by the basement membrane perimeter. For both outcomes, a mean was calculated for each mouse, and values for all mice within a group were then compiled to obtain a mean per group.

Masson's trichrome was used to quantify the content of ASM. All airways cut transversely in three non‐contiguous lung sections were analysed, representing 2–14 airways per mouse (mean and SD of 8.9 ± 2.6). The content of ASM in each airway was calculated by measuring the area occupied by ASM divided by the square of its basement membrane perimeter. A mean was calculated for each mouse, and values of all mice within one group were then compiled to obtain a mean per group.

### 2.9 Data analyses

Two‐way ANOVAs were used to assess the effect of sex, experimental asthma and their interaction on each measured outcome. When the interaction was significant, it was followed by Sidak's multiple comparisons test to compare specifically between sexes in control and asthmatic mice separately. All statistical analyses were performed using Prism 9 (v.9.1.1; GraphPad, San Diego, CA, USA). A value of *P* < 0.05 was considered statistically significant.

## 3 RESULTS

**FIGURE 2:**
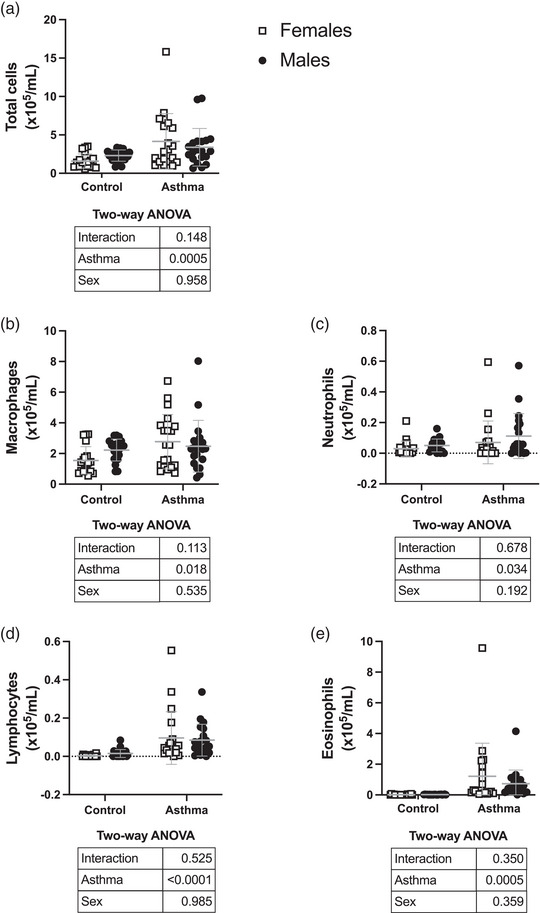
Inflammatory cells in bronchoalveolar lavages. Depicted are: (a) total cells; (b) macrophages; (c) neutrophils; (d) lymphocytes; and (e) eosinophils, all expressed per millilitre of bronchoalveolar lavages. Data are individual results, together with means ± SD for *n* = 20. Results of the two‐way ANOVA are shown in the table below each graph.

Body weight was lower in females than in males (17.85 ± 0.83 and 22.39 ± 1.31 g for females and males, respectively; *P* < 0.0001). Total and differential cell counts per millilitre of BAL are depicted in Figure 2. The HDM increased the total cells (*P* = 0.0005) by a mean of 161% in females (1.6 ± 0.9 × 10^−5^ and 4.2 ± 3.6 × 10^−5^/mL in control and asthmatic mice, respectively) and 47% in males (2.3 ± 0.7 × 10^−5^ and 3.4 ± 2.5 × 10^−5^/mL in control and asthmatic mice, respectively). The HDM also increased all cell types in both sexes by a mean of 79% and 11% for macrophages, 142% and 121% for neutrophils, 2811% and 457% for lymphocytes, and 8192% and 3689% for eosinophils in females and males, respectively. When differential cell counts were expressed as percentages, HDM decreased macrophages (*P* < 0.0001) from 97 ± 3% to 75 ± 16% in females and from 96 ± 2% to 76 ± 13% in males; it increased lymphocytes (*P* < 0.0001) from 0.3 ± 0.3% to 2.2 ± 1.6% in females and from 0.6 ± 0.7% to 2.2 ± 1.3% in males; and it increased eosinophils (*P* < 0.0001) from 0.9 ± 1.3% to 20.4 ± 15.2% in females and from 0.9 ± 0.8% to 18.9 ± 11.4% in males. Sex did not significantly affect the total and differential cell counts, expressed either as the absolute number of cells or as percentages.

**FIGURE 3:**
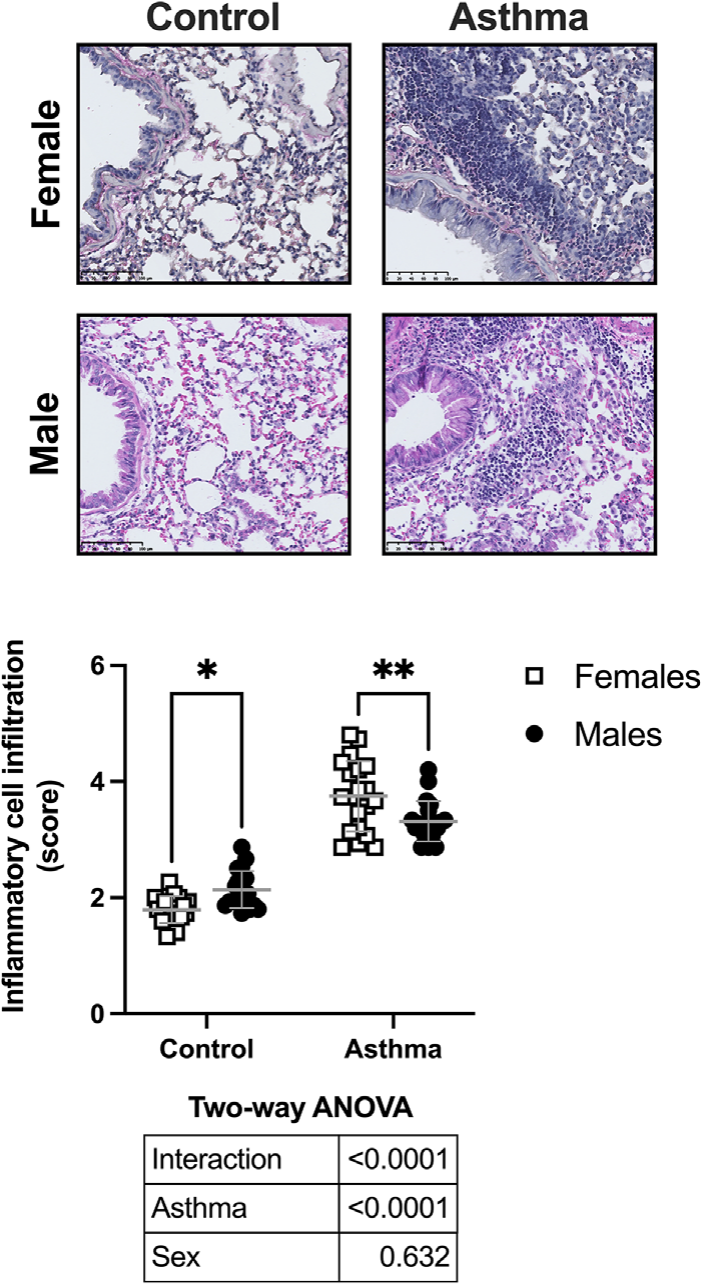
Tissue infiltration with inflammatory cells. Depicted are a representative image for each group and a scatter plot showing the compilation of results. Data are individual results, together with means ± SD for *n* = 20. Results of the two‐way ANOVA are shown in the table below the graph. Asterisks are from post hoc tests showing significant differences (*P < 0.05 and **P < 0.01, respectively).

Tissue infiltration with inflammatory cells is depicted in Figure 3. The HDM increased cellular infiltration (*P* < 0.0001). There was also a significant interaction between sex and asthma (*P* < 0.0001). Post hoc analyses demonstrated that although more cells were found in the lung tissue of male than female control mice (*P* = 0.02), more cells were found in the lung tissue of female than male asthmatic mice (*P* = 0.002).

**FIGURE 4:**
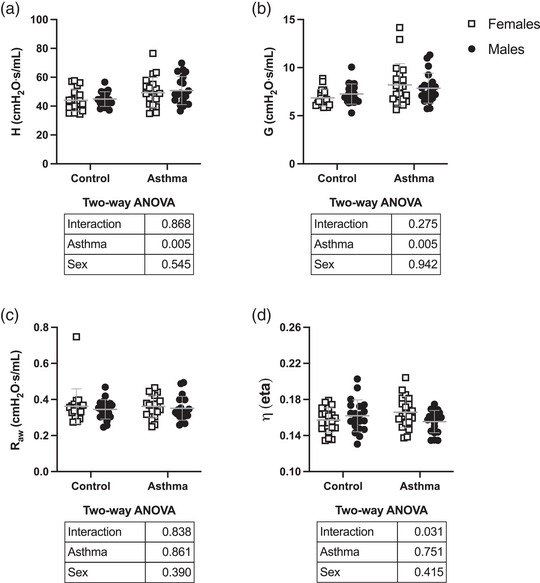
Respiratory mechanics at baseline. Depicted are: (a) lung elastance (*H*); (b) tissue resistance (*G*); (c) airway resistance (*R*_aw_); and (d) hysteresivity (η). Data are individual results, together with means ± SD for *n* = 20. Results of the two‐way ANOVA are shown in the table below each graph.

Respiratory mechanics at baseline (before methacholine challenge) are depicted in Figure 4. The HDM increased lung elastance (*H*; *P* = 0.005) and tissue resistance (*G*; *P* = 0.005), but not airway resistance (*R*_aw_) and hysteresivity (η). Sex had no effect on baseline respiratory mechanics. The only exception was hysteresivity, for which there was a significant interaction between sex and asthma (*P* = 0.03). Indeed, hysteresivity tended to be greater in male than female control mice, but greater in female than male asthmatic mice. However, post hoc analyses indicated that these tendencies were not statistically significant.

**FIGURE 5:**
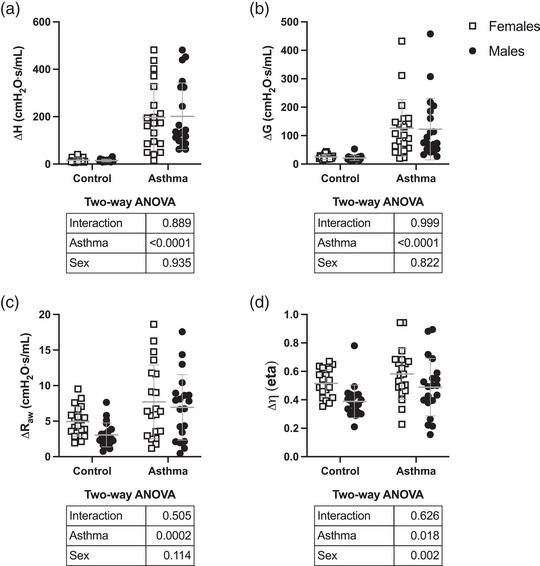
The methacholine response. Depicted are the changes in: (a) lung elastance (Δ*H*); (b) tissue resistance (Δ*G*); (c) airway resistance (Δ*R*_aw_); and (d) hysteresivity (Δη) caused by methacholine (100 mg/mL in females and 50 mg/mL in males). Data are individual results, together with means ± SD for *n* = 20. Results of the two‐way ANOVA are shown in the table below each graph.

The response to methacholine is depicted in Figure 5. The HDM increased the response to methacholine, irrespective of the metric used (Δ*H*, Δ*G*, Δ*R*_aw_ or Δη) to assess the response, confirming hyperresponsiveness to methacholine. There was no effect of sex, or interaction between sex and asthma, for Δ*H*, Δ*G* or Δ*R*_aw_, indicating that the methacholine response was well matched between females and males. According to the experimental design, the comparable increase in Δ*R*_aw_ in females and males also confirmed that the same degree of bronchoconstriction was achieved between sexes. However, sex had a significant effect on hysteresivity (*P* = 0.002), with males showing an attenuated increase in hysteresivity in response to methacholine in comparison to females.

**FIGURE 6:**
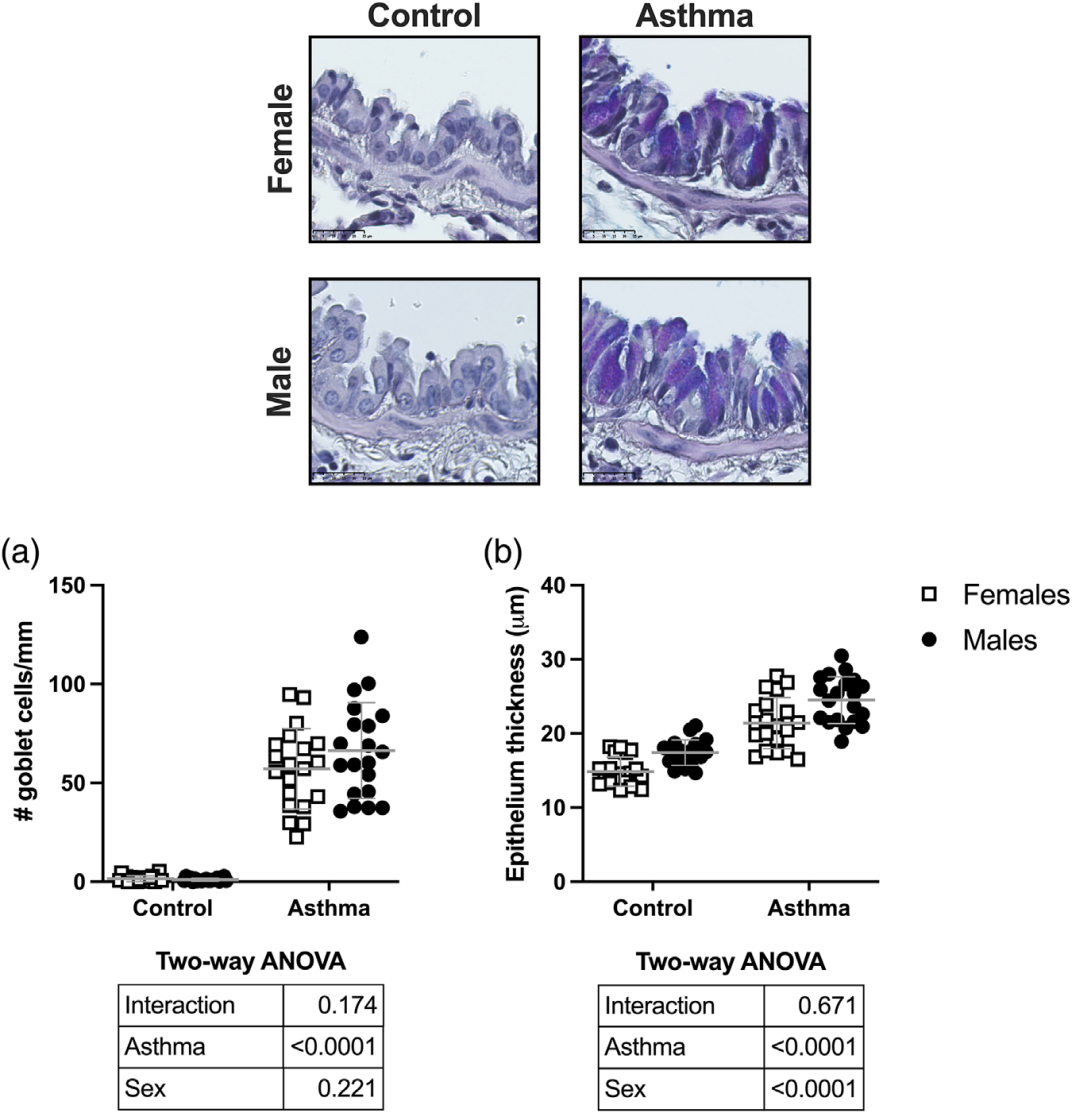
Epithelial metaplasia. Depicted are a representative image for each group and scatter plots showing the compilation of results for: (a) the number of goblet cells per millimetre of basement membrane; and (b) the thickness of the epithelium. Data are individual results, together with means ± SD for *n* = 20. Results of the two‐way ANOVA are shown in the table below each graph.

The number of goblet cells and the epithelial thickness are depicted in Figure 6. The HDM markedly increased the number of goblet cells (*P* < 0.0001). The number of goblet cells and their hyperplasia in response to HDM were not affected by sex. The HDM also increased the thickness of the epithelium (*P* < 0.0001). This thickening occurred to a similar extent between sexes (interaction: *P* = 0.67). Yet, the thickness of the epithelium was affected by sex (*P* < 0.0001), with males showing a thicker epithelium than females.

**FIGURE 7:**
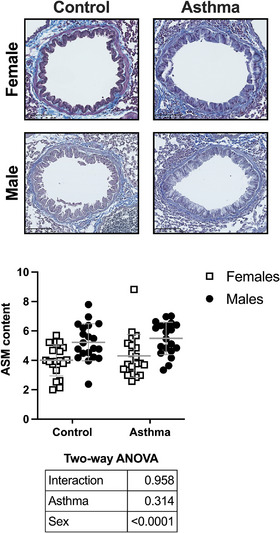
Airway smooth muscle (ASM). Depicted are a representative image for each group and a scatter plot showing the compilation of results for the area occupied by the ASM divided by the square of the airway basement membrane perimeter. Data are individual results, together with means ± SD for *n* = 20. Results of the two‐way ANOVA are shown in the table below the graph.

The content of ASM is depicted in Figure 7. The HDM did not affect the content of ASM. Yet, the ASM content was affected by sex, with males showing a larger amount of ASM than females (*P* < 0.0001). Notably, the size of airways analysed by histology was not different between females and males. Among the 376 female and 418 male airways displaying a full circumference, the average perimeter was 0.964 ± 0.497 and 0.904 ± 0.509 mm, respectively (*P* = 0.09).

## 4 DISCUSSION

In contrast to previous studies, the extent of pulmonary allergic inflammation was not extensively different between sexes. In fact, total and differential cell counts in BAL were not statistically significant. One exception was the infiltration of inflammatory cells within the lung tissue, which was greater in male control mice and greater in female asthmatic mice. Baseline mechanics were also very similar between sexes. The only exception was hysteresivity, which was modulated differently by asthma in females versus males. Lung stiffening and bronchoconstriction caused by methacholine were also comparable between sexes, although this matched response was triggered by half the dose in males compared with females. Again, the only exception was hysteresivity. The methacholine‐induced change in hysteresivity was significantly less in males than females. In terms of airway wall remodelling, both sexes exhibited a substantial and comparable increase in goblet cells in the epithelium. The thickening of the epithelium caused by HDM was also similar between sexes, although males had a thicker epithelium. Finally, HDM did not increase the content of ASM within the airway wall, although males had more ASM than females. Together, these results provide further insights into the mechanisms underlying sex disparities in mouse models of asthma.

Females are reputed to be more susceptible than males to the development of pulmonary allergic inflammation (E‐Lacerda et al., 2020; Hayashi et al., 2003; Laffont et al., 2017; Lauzon‐Joset et al., 2020; Melgert et al., 2010, 2005; Mostafa et al., 2022; Schaefer et al., 2020; Seymour et al., 2002; Takeda et al., 2013; Weiss et al., 2021; Zhao et al., 2019). Although this was not the focus of the present study, we confirmed these results somewhat, by showing a greater infiltration of inflammatory cells in the lung tissue of female mice. This sex difference was rather small, however. It was also restricted to the tissue compartment, because only trended differences were seen between sexes in BAL. Given that females were smaller than males in the present study, the greater inflammatory response in the former might also have been caused by a relatively higher dose of HDM.

Another obvious airway wall alteration in acute murine models of asthma is the metaplasia of the epithelium, characterized by goblet cell hyperplasia (Evans et al., 2015). This feature was observed clearly in our study. The epithelium was also thicker in HDM‐exposed mice. The extent to which both these features occurred was similar between sexes. Surprisingly, however, the epithelium was inherently thinner in females. To our knowledge, this has never been reported before. The functional consequence of a thinner epithelium is unknown. It is tempting to speculate that it might facilitate sensitization to inhaled allergens and, thereby, contribute to the increased susceptibility of females to the development of pulmonary allergic inflammation. More studies will be needed.

Respiratory mechanics at baseline (before methacholine) were also altered to a similar extent in both sexes by experimental asthma. It was characterized by increases in lung elastance (*H*) and tissue resistance (*G*). These results might appear inconsistent with our previous study (Boucher, Henry, Khadangi et al., 2021), in which only *H* was sightly but significantly increased by asthma in female mice. These inconsistencies are attributed to the presence (vs. the absence) of deep inflations before the measurement of respiratory mechanics. In contrast to our previous study, we did not use deep inflations in the present study. Deep inflations are recruitment manoeuvres (Reiss et al., 2011; Robichaud et al., 2017); viz., they are recruiting part of the lung that is otherwise not reachable from the input oscillatory flow owing to airway closure. These recruitment manoeuvres are normally recommended because they prevent lung injury during long protocols of mechanical ventilation (Reiss et al., 2011). They also decrease variability between subjects by eliminating factors that influence respiratory mechanics, such as airway closure (Robichaud et al., 2017). This does not only increase statistical power, it also ascertains that the observed difference between groups is attributable to an intrinsic change in the mechanical properties of the lung tissue, and not to airway closure. Yet, airway closure is a prominent feature of asthma (Chapman et al., 2008; Dame Carroll et al., 2015; Farrow et al., 2012; King et al., 1998; Nilsen et al., 2020; Wagers et al., 2004). Thus, the counterargument is that these recruitment manoeuvres should be avoided because they eliminate important mechanical differences between control and asthmatic mice. The fact that asthma in the present study increased *H* and *G*, which are both sensitive to closure, while leaving airway resistance (*R*_aw_) unchanged supports the contribution of airway closure. The fact that the increased *H* and *G* caused by experimental asthma were almost completely eliminated with recruitment manoeuvres in our previous study (Boucher, Henry, Dufour‐Mailhot et al., 2021) also confirms that the main mechanical alteration seen at baseline is attributable to small airway closure.

Interestingly, there was a significant interaction between asthma and sex for baseline hysteresivity. Hysteresivity is the ratio of *G* over *H*. It was first promoted as a complementary metric because, theoretically, it should not be affected by the amount of tissue being probed but solely by the mechanical properties of the tissue being probed (Fredberg & Stamenovic, 1989). For example, closure decreases the amount of tissue being probed without affecting its mechanical properties. It should thus keep hysteresivity unchanged even if it inevitably increases both *G* and *H*. In practice, however, *G* was shown to be very sensitive to airway narrowing heterogeneity (Lutchen et al., 1996). As a result, an increased in hysteresivity in response to an intervention (induced inflammation or a methacholine challenge) is now considered an index of airway narrowing heterogeneity (Bates et al., 2009). In our study, the observed significant interaction between asthma and sex thus suggested that airway narrowing heterogeneity is affected differently by asthma in females versus males. This sex disparity was small, however, because post hoc tests indicated no statistically significant differences between sexes in both control and asthmatic mice.

The response to methacholine was then investigated. As aforementioned, it is firmly established that males are more responsive to methacholine than females (Boucher, Dufour‐Mailhot et al., 2022; Card et al., 2006, 2007). There was no motive to show this sex disparity again. We therefore tried to match the degree of bronchoconstriction between sexes to determine the relative weights of the different mechanisms contributing to the methacholine response. Using oscillometry and computational models, it is possible to dissect out the underlying contributors to the methacholine response (Bates et al., 2011). As outlined above, a concomitant increase in *H* and *G* is an indicator of lung stiffening, occurring as a result of a stiffening of the lung tissue and/or small airway closure. Other aforementioned examples are an increased in *R*_aw_, which is an indicator of bronchoconstriction, and a disproportionally larger increase in *G* than in *H*, which is an indicator of airway narrowing heterogeneity. In the present study, we observed a similar methacholine response between sexes in terms of Δ*H*, Δ*G* and Δ*R*_aw_ using a dose of methacholine twice as high in females. This confirmed indirectly that males are more responsive than females. It also suggested that lung stiffening and bronchoconstriction caused by methacholine did occur to the same extent between sexes. Surprisingly, however, the gain in hysteresivity caused by methacholine (the indicator of airway narrowing heterogeneity) was markedly attenuated in males. Thus, these results suggested that for a matched methacholine response in terms of lung stiffening and bronchoconstriction, males exhibit less airway narrowing heterogeneity. This might be important because airway narrowing heterogeneity is one of the main contributors to hyperresponsiveness in asthma (Dame Carroll et al., 2015; Downie et al., 2007; Farrow et al., 2017; Hardaker et al., 2011; King et al., 2004; Lutchen et al., 1996; Petak et al., 1997).

Finally, we measured the content of ASM within the airway wall. In both sexes, experimental asthma did not affect the content of ASM. This might not be surprising given the acute nature of the HDM model used in the present study. Interestingly, however, males had inherently more ASM than females. According to our knowledge, this has never been reported. This observation will need to be reproduced. It is a structural feature that might be of great functional importance (Lambert et al., 1993). For instance, it might contribute to the enhanced methacholine response seen in males. Perhaps it also contributed to the attenuated methacholine‐induced airway narrowing heterogeneity. In support of this conjecture, we have demonstrated recently that although an intervention enhancing the contractile capacity of the ASM translates effectively into an increased methacholine response in terms of Δ*H*, Δ*G* and Δ*R*_aw_, it concurrently attenuates airway narrowing heterogeneity (Boucher, Dufour‐Mailhot et al., 2022).

Notably, a thicker epithelium might also contribute to hyperresponsiveness. This is attributable to a geometric effect (Bossé, 2021); i.e., with an increased amount of material internal to the ASM (thicker lamina propria, epithelium and/or airway lining fluid), any given degree of ASM shortening translates into a greater degree of luminal constriction. Thus, both thicker epithelium and an increased amount of ASM might contribute to hyperresponsiveness in males. It is, in fact, more likely that hyperresponsiveness emerges from the agglomeration of potentiating factors acting synergistically (Bates, 2016). Many molecular alterations might also be at the origin of this greater response to methacholine in males versus females. More studies in this area are clearly warranted.

## 5 CONCLUSION

In an acute mouse model of asthma induced by repeated intranasal exposures to HDM, both sexes exhibited all the expected features of experimental asthma, including infiltration of inflammatory cells into the lung, airway epithelial metaplasia and hyperresponsiveness to methacholine. Therefore, both sexes are susceptible and can thus be used in future studies, ideally together for purposes of comparison. The most striking difference between sexes remains the greater degree of responsiveness to methacholine in males. The increased amount of ASM in males compared with females, which is reported herein for the first time, provides a likely explanation for this sex disparity. The data also suggested that a more muscular airway tree might protect against airway narrowing heterogeneity, which might be important functionally to maintain a proper alveolar ventilation in lung diseases showing an increased propensity for airway narrowing heterogeneity and closure, such as asthma (Chapman et al., 2008; Dame Carroll et al., 2015; Downie et al., 2007; Farrow et al., 2012, 2017; Hardaker et al., 2011; King et al., 2004, 1998; Lutchen et al., 1996; Nilsen et al., 2020; Petak et al., 1997; Wagers et al., 2004). More studies are warranted to dismantle this yin and yang effect of the ASM and to untangle how it plays out in asthma.

## AUTHOR CONTRIBUTIONS

Magali Boucher and Cyndi Henry contributed to the development of the experimental design, analysed the data and performed laboratory experiments. Rebecka Gill and Andrés Rojas‐Ruiz performed laboratory experiments. Ynuk Bossé contributed to the development of the experimental design, analysed the data and wrote the manuscript. All authors edited the manuscript, approved the final manuscript and agree to be accountable for all aspects of the work in ensuring that questions related to the accuracy or integrity of any part of the work are appropriately investigated and resolved. All persons designated as authors qualify for authorship, and all those who qualify for authorship are listed.

## CONFLICT OF INTEREST

None declared.

## Data Availability

The datasets used and analysed during this study are available from the corresponding author on reasonable request.
